# Developmental trauma: Conceptual framework, associated risks and comorbidities, and evaluation and treatment

**DOI:** 10.3389/fpsyt.2022.800687

**Published:** 2022-07-22

**Authors:** Daniel Cruz, Matthew Lichten, Kevin Berg, Preethi George

**Affiliations:** ^1^Hackensack Meridian Health Mountainside Medical Center, Montclair, NJ, United States; ^2^Independent Private Practice, Clifton, NJ, United States

**Keywords:** developmental trauma disorder, complex trauma, adverse childhood experiences, PTSD, Bronfenbrenner's

## Abstract

Children exposed to adverse childhood experiences (ACEs) and pervasive interpersonal traumas may go on to develop PTSD and, in most cases, will further undergo a significant shift in their developmental trajectory. This paper examines contemporary research on Developmental Trauma (DT), which is inextricably linked to disruptions in social cognition, physiological and behavioral regulation, and parent-child attachments. Developmental trauma associated with early experiences of abuse or neglect leads to multi-faceted and longstanding consequences and underscores critical periods of development, complex stress-mediated adaptations, and multilevel, trans-theoretical influences in the diagnostic formulation and treatment of traumatized children, adolescents, and adults. Psychological and medical correlates of Developmental Trauma Disorder are considered, and directions for future research are discussed.

There has been much interest in understanding the prevalence and impact of adverse childhood experiences (ACEs), which refer to potentially traumatic events that occur in childhood and adolescence (e.g., abuse, rejection, and abandonment by caregivers; loss of a caregiver; interpersonal violence exposure). Recent developments suggest that multiple, repeated experiences of ACEs and interpersonal traumas have broad, cumulative, and lasting effects [e.g., (1, 2)]. Children are more likely than adults to lack the cognitive and behavioral capacities to understand and respond to traumatic circumstances effectively. Children affected by interpersonal trauma often experience more global and profound changes than adults who conceivably have more developed adaptations to stress and more cognitive resources to mitigate risks and promote resiliency. The current article provides discussion of how early, repeated interpersonal traumas can interrupt the development of secure attachment and precipitate the emergence of chronic and severe traumatic adaptations, followed by an analysis of contemporary research, conceptual and diagnostic issues, and assessment and treatment. The paper concludes with a discussion about future directions for research and practice.

## Trauma and PTSD

Children and adults who have experienced trauma and developed measurable mental health symptoms associated with the trauma may present with indications of posttraumatic stress disorder (PTSD) that warrant careful diagnostic evaluation. PTSD is a psychiatric disorder brought on by exposure to a highly stressful and potentially life-threatening event such as a natural disaster, motor vehicle accident, witnessing family or community violence, experiencing abuse and neglect, or losing a loved one. The symptoms of PTSD, as defined by the fifth version of the Diagnostic and Statistical Manual of Mental Disorders (DSM-5) criteria, are characterized by persistent and intrusive thoughts, hyperarousal (i.e., heightened startle in response to unexpected sounds or movements), deliberate avoidance of trauma reminders, and alterations to conscious awareness (i.e., dissociation, derealization, and depersonalization). Persistent trauma symptoms (i.e., that last longer than a month after the traumatic event(s) and are accompanied by social, behavioral, and academic impairments) indicate the presence of PTSD and differentiate it from other psychiatric disturbances [e.g., (3)]. Healthcare providers widely use the DSM-5 to determine whether a person's symptoms are severe enough to warrant a diagnosis of PTSD.

However, individuals exposed to ACEs, pervasive interpersonal traumas, and polyvictimization [multiple, repeated ACEs; (4–8)] may not only go on to develop PTSD, but may also undergo significant shifts in their developmental trajectories. The changes are often widespread, adversely affecting their biological, social, cognitive, emotional, and spiritual/existential development (1, 9–11). Moreover, these experiences tend to threaten core beliefs and assumptions, including their self-esteem as well as their sense of lovability, vulnerability, and faith in family, friendships, and a higher power. Trauma survivors often face lifelong challenges in developing and maintaining trusting relationships; building and utilizing healthy coping strategies; and adjusting to school and, eventually, the workplace (12–14). For example, ACEs positively predict PTSD, recurrent depression, and repeated suicide attempts (15–17), and findings from several meta-analyses consistently link ACEs to enduring and debilitating psychiatric and physical health disorders (1, 18). It has been suggested that individuals who have experienced ACEs often view the world as unfair and to blame for their circumstances, resulting in reoccurring helplessness and hopelessness about life and relationships, including their ability to make a positive and meaningful impact. Indeed, retrospective studies with adults reveal that 50–60% of adults have a history of childhood abuse or neglect that has impacted their emotional development, their core assumptions about themselves and their worlds, and their functioning as adults (19, 20).

### Children and PTSD

Research suggests that children and adolescents who experienced significant early interpersonal trauma often receive inaccurate or incomplete diagnoses later in life [thereby leading to various treatments for co-occurring psychiatric disorders that may not be effective; (21, 22)]. Scholars spanning a broad range of scientific disciplines have asserted that this is due to significant limitations in the DSM's PTSD classification system (23–28). Repeatedly traumatized children and adolescents share overlapping symptoms of PTSD; however, experiences of prolonged trauma during sensitive periods of development are more detrimental to children, given their age, limited cognitive capacities, and dependency on caregivers. A new conceptualization of trauma—specifically the construct of complex trauma—was developed for repeatedly traumatized children and adolescents who often have high comorbidities between trauma and other disorders, which are underestimated by PTSD diagnostic criteria (29–31).

From this perspective, the diagnosis of PTSD only accounts for a limited portion of the symptoms experienced by individuals who have experienced childhood trauma. These individuals often demonstrate broader and more severe health problems, social and economic adversity (including poverty and homelessness, psychiatric hospitalization, incarceration), and medical and psychiatric comorbidities as well as persistent negative views of themselves, others, and their life circumstances (32–34). In addition, they may re-enact their traumas through defensive (protective) expressions of anger and aggression (because they fear emotional vulnerability and experiencing additional traumas) and by coping with alcohol, drugs, and self-inflicted injuries to numb their emotional pain and suffering (35).

The differing presentations of trauma-related disorders that span a wide range of medical and psychological comorbidities frequently go unnoticed with classical PTSD diagnostic approaches, and, therefore, are often underreported and, in turn, untreated (36). Individuals who have experienced prolonged, repeated interpersonal traumas often present with overlapping symptoms of PTSD and a multiplicity of mental health conditions such as attention deficit hyperactive disorder (ADHD, including late-onset in adulthood), autism spectrum disorders (ASD, including social-communication disturbances), psychosis, and mood and personality disorders (37–42).

### Complex post-traumatic stress disorder

The conceptualization of complex trauma emphasizes prolonged and unavoidable circumstances, regardless of one's age. Compared to those with PTSD, patients with complex PTSD (c-PTSD) consistently report cumulative traumas, including interpersonal violence exposure and sexual abuse (30, 43–50). c-PTSD has been studied extensively in diverse samples, including racial and sexual minorities as well as human trafficking victims who have experienced repeated physical and sexual assault (51–54). Nevertheless, more studies of children with c-PTSD are needed [i.e., (55)].

Adolescents and adults who have experienced trauma and developed measurable mental health symptoms, as a result, may present with indications of c-PTSD that warrant careful diagnostic evaluation. As defined by ICD-11 criteria, the symptoms of c-PTSD are characterized by three PTSD symptom clusters: (1) persistent and intrusive thoughts, (2) hyperarousal (i.e., heightened startle in response to unexpected sounds or movements), and (3) deliberate avoidance of trauma reminders. c-PTSD is differentiated by a second higher-order measurement factor, characterized by disturbances in self-organization (DSO), which encompasses affective dysregulation, negative self-concept, and pervasive disturbances in relationships (56–58).

DSO is also highly prevalent in patients with borderline personality disorder [BPD; (59)]. More specifically, c-PTSD and BPD have overlapping symptoms of anxiety sensitivity, low psychological distress tolerance, dissociation, and relational problems originating from attachment insecurities and, in clinical contexts, parataxic distortions whereby patients re-enact interpersonal behaviors with healthcare providers that mirror past relationships (60–63). However, empirical findings suggest that BPD only accounts for a limited portion of the symptoms reported by individuals who have experienced childhood trauma and that characteristics of c-PTSD and BPD correlate with, and are distinct from, several other psychiatric disorders such as unipolar and bipolar depression, substance abuse, and functional neurological disorders [e.g., (59, 64–68)].

## Developmental trauma: An overview

Developmental Trauma (DT) refers to the complex and pervasive exposure to life-threatening events that (1) occurs through sensitive periods of infant and child development, (2) disrupts interpersonal attachments, (3) compromises an individual's safety and security operations, (4) alters foundational capacities for cognitive, behavioral, and emotional control, and (5) often contributes to the development of complex PTSD in adulthood (69–74). DTD emerges from prolonged and cumulative interpersonal trauma that disrupts the development of secure attachments to caregivers and dramatically alters core assumptions and beliefs about one's vulnerability to danger in the world. DTD is theorized to develop from early interpersonal trauma. However, DTD may also result from the lack of a secure attachment relationship, which would have protected the developing individual during early trauma events. For example, a child who, within the context of a securely attached relationship with their caregiver, is exposed to abuse is likely to fare more positively than one who is exposed to the same abuse, yet lacks a secure attachment. The diagnosis of DTD was proposed for inclusion in the DSM-5 as an alternative to PTSD to better address the timing in which traumatic events occur (e.g., during sensitive periods of brain development) and the impacts on children's self-regulation skills and relational capacities (75). DTD attempts to provide this differentiation by underscoring experiences of pervasive, complex traumas that occurred early in life for children, and for adults whose physical, psychological, social and emotional disturbances originated from these experiences, which may be repeatedly interfering with their relationships, quality of life, self-identity, and life satisfaction.

DT has been postulated to result in symptoms that extend beyond those of PTSD and that often occur when traumatized children and adolescents are exposed to developmental trauma(s). These symptoms may complicate or, in some cases, account for the mental health problems that lead to the diagnosis of several childhood/adolescent psychiatric disorders. This has been confirmed in a number of large-scale field trials, cross-sectional and longitudinal investigations, and comparative studies spanning diverse psychiatric groups (24, 31, 76, 77). It has been suggested that DTD is set apart from PTSD in that the former is precipitated by toxic stress, referring to prolonged activation of the stress response system, particularly the hypothalamic-pituitary-adrenal (HPA) axis, in the absence of treatments and supportive peers and adults (78, 79). In addition, attachment disturbances associated with DTD originate at the *beginning* of an individual's lifespan development (e.g., parental neglect and abandonment, parents addicted to alcohol and drugs, separation from caregivers. By comparison, the causal events that lead to PTSD can occur at any point (or points) in one's life cycle.

Investigations of individuals with DTD suggest that developmental trauma is often characterized by (1) poor self-identity development, (2) interpersonal sensitivity and consistent problems in relationships, including with peers, adults, and primary caregivers, (3) high rates of exposure to family and community violence, (4) high rates of psychiatric comorbidities, and (5) chronic and debilitating medical/neurological illnesses (24, 28, 34, 36, 80–82). Indeed, several studies have confirmed high comorbidities between PTSD and other disorders including depression, anxiety and panic/agoraphobia, psychotic disorders, and functional medical syndromes that might warrant differentiated assessment procedures and treatments (83–85). Studies of children living in pervasively abusive and neglectful circumstances indicate that children with characteristics of DTD commonly (often exclusively) rely on behavioral inhibition and cognitive dissociation; these responses are mediated by conditioned fear and physiological hypoarousal [i.e., the freezing adaptation of the stress response system; (86)].

Numerous studies suggest that individuals with complex traumas and DTD tend to cope with their traumas by deliberately evading reminders of the original trauma and by denying that such events occurred, including on self-report trauma symptom measures (87, 88), thereby conferring additive risks (i.e., dissociation and traumatic amnesia, undermining mental health needs). These traumatic stress adaptations, in turn, can contribute to experiences of emotional numbing and low frustration tolerance and precipitate behavioral outbursts that result in refractory (treatment-resistant) mood disturbances, long-term psychiatric hospitalizations (and re-hospitalizations), removal from school, and placement in foster care and juvenile justice treatment centers (89–93).

The DSM-5 indicates that the sequelae of “prolonged, repeated, and severe traumatic events (e.g., child abuse)” include symptoms that are consistent with DTD: “difficulties in regulating emotions or maintaining stable interpersonal relationships, or dissociative symptoms” (p. 276). However, there is vigorous debate regarding the viability of this alternative designation, given that the criteria for other specified trauma and stress-related disorders are broad, lack specificity, include interpersonal and non-interpersonal antecedents, and require that providers first consider alternative-mainstream taxonomies. For example, DTD must be differentiated from PTSD, reactive and disinhibited attachment disturbances, and borderline personality disorder, the latter of which is marked by pervasive mood instability, impaired self-identity, dissociation, and provocative and confrontational interpersonal behaviors [often at extremes of the idealization and devaluation continuum; (60, 66, 68, 94)]. As such, several different approaches are used to diagnose persons who have experienced early, recurring stressors and are displaying indications of developmental trauma disorder.

## Developmental trauma: Diagnostic issues

Researchers have developed objective procedures to diagnose children with indications of DTD (e.g., standardized interviews and psychometric instruments specifically designed to assess for trauma-mediated interpersonal and attachment disturbances). The Developmental Trauma Disorder - Semi-structured Interview (DTD-SI) is one such tool. DTD expert consensus guidelines and psychometric studies on the validity and reliability of the DTD criteria and the DTD-SI suggest that three factors differentiate DTD from other psychiatric disorders, including PTSD (24, 28, 34, 95, 96). These factors include emotion and physiological dysregulation (e.g., alexithymia, sensitivity to touch, functional neurological disorders), cognitive processing and behavioral problems (e.g., traumatic re-enactments, self-mutilation, dissociation, impaired attention, learning, memory), and poor self-other awareness (characterized by low self-esteem and self-hatred, insecure attachment, experiences of interpersonal betrayal and persistent states of anger, resentment, and revenge).

### PTSD preschool adaptation

Another approach to diagnosing children with trauma, especially for those who fail to meet the criteria for PTSD, is to revise the requirements of the DSM so that it is more developmentally sensitive to children. The American Psychiatric Association conferred a preschool subtype PTSD diagnosis for children between the ages of 0–6, as an alternative to the standard PTSD classification that originated from work with adults (3). The alternative set of criteria used for the preschool subtype PTSD includes several changes to the standard DSM PTSD criteria, ranging from adjustments to clinical thresholds (e.g., lowered from three to one avoidance symptom) to the removal of items deemed inappropriate for children (e.g., expression of immediate distress following the trauma; nightmares that necessarily include traumatic content; a sense of a foreshortened future, which requires complex cognitive skills). DSM-5 criteria for the preschool PTSD subtype require that the “duration of the disturbance [referring to trauma symptoms] is more than 1 month” (3, p. 273). The DSM preschool subtype is also more developmentally sensitive to children in that it emphasizes symptoms of trauma that are unique to younger individuals (e.g., traumatic reenactments in interactions with peers during playtime) and focuses more on trauma that is particularly relevant for children, such as harm to the wellbeing of caregivers or disruptions to the quality of the parent-child relationship (e.g., having a caregiver who is medically ill and unable to care for the child, foster care placement, loss of a primary caregiver). These revised criteria were designed with both assessment and treatment in mind, underscoring the recognition that symptoms of PTSD may naturally present themselves in children's play behaviors and not necessarily in their verbal responses to the myriad of questions posed by assessors in standard clinical interviews. This revised DSM-5 taxonomy conceptualizes children with trauma as a single (or homogenous) group. In other words, it implies that children with trauma differ from adults with trauma and, at the same time, that they reliably differ from children who have not experienced trauma (or do not present with clinical symptoms as a result of trauma). Studies suggest that providers can identify and treat traumatized children more effectively using this revised classification (97–99).

## The development of DTD: Bronfenbrenner's multiple levels of analysis framework

As a multifactorial neurobehavioral disturbance, developmental trauma causes significant alterations to children's cognitive, emotional, physiological, and relational capacities, and as a result, they experience widespread disruptions to their academic, social, and occupational functioning (100–102). At the same time, mental health disturbances linked to the development of trauma are mediated and/or moderated by a broad range of factors, all of which have the potential to mitigate the longitudinal course of developmental trauma, associated psychiatric comorbidities, and functional impairments. This notion is supported by Bronfenbrenner's Socio-Ecological Model [SEM; (103)], which asserts that multiple levels of influence, specifically individual, interpersonal, organizational, community, and public policies, are needed to understand the diverse range of adaptations associated with interpersonal trauma, in that they can confer additive risks or, conversely, potentiate positive and resilient transformations in response to adverse childhood experiences.

The physiological, attachment, and meta-cognitive disturbances seen in traumatized children intersect at multiple levels of Bronfenbrenner's model. More specifically, children and adolescents who have experienced severe trauma and ACEs experience high rates of poverty, legal problems, and violence exposure (89, 104–106). Abusive parents often have experienced their own traumas and attachment disturbances (107–109), which are often compounded by high rates of substance abuse, depression, financial stress, and unemployment (12, 110–113). Racial, ethnic, sexual, and neurodiverse minorities are affected by developmental trauma at very high rates (114, 115), suggesting that systemic factors such as state and national policies, allocation of resources for prevention and intervention, discrimination, and stigma exert influence over, and significantly impact responses to, trauma and recovery.

Children with trauma live in persistent states of fear and terror, and recurring traumas become transformative developmental experiences that alter their global appraisals and future responses to stress (14, 49, 61, 116). This heightened fear propensity disrupts self-awareness, information processing, interpersonal communication, and mastery of age-appropriate developmental competencies [e.g., establishing healthy relationship parameters, identifying and expressing feelings appropriately, and tolerating ambiguity; (43, 90)] and causes individuals to have problems learning, making friends at school, and appreciating their strengths and unique abilities, which are often overshadowed by their social and behavioral difficulties.

Researchers have described several adverse physiological alterations underlying cognitive disturbances in children who have experienced early traumas (13, 117–124). These include, for example, stress-mediated changes to the organization and functioning of the amygdala, hippocampus, and prefrontal cortex, which exert influence over fear processing, including automatic (impulsive) behavioral responses to perceived threats (125–130), learning and memory [fear memories override attention, concentration, and, in turn, inhibit learning of new information; (72, 131, 132)], and higher-order (complex) cognitive operations [e.g., problem-solving and learning from experience, stress appraisal and reappraisal, perceived controllability; (133–135)]. As an illustration of this, reinforcement and contingency learning studies indicate that children who had experienced severe adversity or developmental trauma are not able to adjust their behavior effectively to shifting environments or demands, regardless of threatening and non-threatening circumstances (136, 137). These findings suggest that individuals who have endured significant stress in their lives, especially during early childhood or adolescence, are at increased risk of experiencing avoidance, anger, frustration, and anxiety as primary ways of being in the world, regardless of their circumstances (138–140). These types of responses can be damaging to an individual's development and ability to function, particularly if their safety needs are undermined. Repeatedly traumatized children and adolescents become habituated to evaluating their surroundings for indications of threat that might render them vulnerable to additional traumas, given that they had limited opportunities to recover from the early and repeated abuse and neglect.

Researchers speculate that the mechanisms involved in the development of traumatic stress adaptations correlate with diverse, interrelated psychiatric symptoms and comorbidities often reported in studies of individuals who have experienced repeated abuse and neglect. This notion is consistent with a socio-ecological framework, the developmental trauma diagnosis, and trans-theoretical models that focus on far-reaching (cross-diagnostic) effects from critical and sensitive periods, attachment and social cognition (e.g., mentalizing and perspective taking), sleep, physiological stress, systemic inflammation, and mood and personality (141–143).

### The role of attachment

Prolonged and cumulative interpersonal trauma disrupts the development of secure attachments to caregivers. According to attachment theorists, securely attached infants seek proximity to their caregivers to minimize anxiety and to form goal-directed partnerships, and/or interactional synchrony, where they mirror each other's communication patterns and learn to anticipate, express, and meet each other's needs. Through these interactions, children develop internal working models, which encompass individuals' core beliefs about their self-worth and lovability as well as their general expectations of others in relationships (144). Insecure attachment styles (e.g., anxious, avoidant, and disorganized) are frequently associated with interpersonal trauma (145–147). Studies have consistently linked attachment disturbances to the emergence (and maintenance) of developmental trauma symptoms, which may remain latent until interpersonal interactions later in life (e.g., peer conflicts, inadequate institutional responses to trauma, workplace challenges) give rise to memories of early trauma (148). The combination of repeated childhood trauma and the absence of parental nurturing, support, and protection (e.g., parents who are addicted to alcohol and/or drugs, are homeless, or are living in severe poverty; family violence; parental incarceration) can be particularly devastating and can potentiate DT (34, 145, 149, 150).

### Social cognition

Cognitive science researchers have expanded upon attachment concepts to include the construct of mentalizing, which is defined as the capacity to consider the mental states of others (e.g., inferring their needs, goals, interests, and intersubjective experiences) as distinct from one's own. Mentalizing has been described in the literature as reflective functioning, theory of mind, metacognition, insight, and perspective-taking (151). Mentalizing deficits have been reported in several clinical populations, including individuals diagnosed with borderline personality disorder, schizophrenia, autism, and more recently, developmental traumas (152–155).

Children and adolescents with DT demonstrate impaired mentalizing (i.e., reflecting functioning), referring to the capacity to effectively understand oneself (needs, strengths, and limitations) and others (e.g., that others may have worldviews that differ from one's own). Early interpersonal trauma and insecure attachment correlate with low reflective functioning (154, 156–158).

Scholars have surmised that these children are likely dissociating (and therefore undermining their intra/interpersonal awareness), fearful and anxious, and preoccupied with maintaining compulsive and highly ritualistic behaviors (41, 159). For example, in response to sexual trauma, victims often report experiencing contamination anxiety such as body shame and disgust and overwhelming fear, that being near others will result in contact contamination (160–162). These individuals, in turn, engage in social avoidance, excessive cleansing, handwashing, and relentless reassurance-seeking to make sure they don't increase the likelihood of illness or misfortune in others.

### Executive functions and self-regulation capacities

Executive functions refer to diverse cognitive abilities including working memory, mental flexibility, and information processing that regulate attention, mood, and behavior; enable adaptive, goal-focused actions; and are instrumental to effective coping, problem solving, and success in school, work, and relationships (163). Traumatic stress is known to cause measurable adverse effects on executive functions (121, 134, 164). For example, recent studies, including several meta-analyses, have found that trauma negatively correlates with working memory, inhibition, and mental flexibility; the adverse effects are often higher for children exposed to multiple traumas compared to those reporting isolated events (93, 165, 166). These executive functioning challenges often result in significant shifts in mood and energy that interfere with concentration, decision-making, and social-interpersonal functioning. Children and adolescents with symptoms consistent with DTD whose symptoms include reckless, disinhibited, and destructive behaviors are likely to have multiple and intersecting psychiatric disturbances, such as conduct disorder and antisocial personality traits, which are known to predict high incarceration rates and recidivism. In turn, these children may grow up feeling stigmatized, unprotected, and personally flawed, which further perpetuates their depression, anger, low self-esteem, and existential despair as well as their increased risk of learning disorders, vocational problems, and criminal records (12, 13, 167, 168).

## Clinical assessment

Patients with DT present with signs of mood disturbances (e.g., poor frustration tolerance, emotional numbing, anger, rage), behavior disturbances (e.g., inflexible routines, self-injurious behaviors, impulsivity), and cognitive disturbances (e.g., inattention, disorganized thinking, poor problem solving), as well as sensory processing problems such as sensitivity to sound and touch as well as limited time and body awareness (169, 170). They often show signs of a heightened startle reflex, dissociation, fear, and anxiety and are often unable to be soothed by others (171–173). For example, studies of children who have experienced trauma and polyvictimization suggest that these children display greater physiological reactivity to threats such as traumatic reminders than children who have not experienced trauma (174–177).

### Clinical signs and symptoms of DTD

Children and adolescents with DTD may react with either over-modulated or under-modulated behaviors. Over-modulated children appear hyperactive and aggressive, whereas under-modulated children appear depressed, withdrawn, and possibly dissociative. Children and adolescents with DTD may be highly reactive to their environment (e.g., hypersensitive to sounds, touch, lights) or appear to be relatively indifferent to their immediate surroundings (80, 178). They may react to perceived threats with avoidance, tantrums, or anger, especially when they feel emotionally vulnerable or disempowered.

When children or adolescents with symptoms consistent with DTD appear distractible, impulsive, and/or highly aggressive, these children are likely to be classified with ADHD (hyperactive and impulsive or combined subtype), conduct disorder, bipolar disorder, and/or oppositional defiant disorder (179–182). These individuals may indeed meet diagnostic criteria for several of these psychiatric disorders, however, they may be secondary to DTD, and, as such, require different clinical evaluation and treatment approaches.

On the other hand, individuals with DTD who are anxious, withdrawn, dissociative, and rigid (adhering to strict behavioral routines) are more likely to be diagnosed (and sometimes misdiagnosed) with neurodevelopmental conditions such as autism spectrum disorders (ASD) because trauma symptoms such as emotional numbing, difficulty socializing with others, obsessive-compulsive behaviors, poor mentalizing (perspective taking), and poor verbal expression mirror characteristics of children with ASD (39, 41). Likewise, studies suggest that, when controlling for comorbid PTSD, individuals with DTD have higher rates of ADHD, social and separation anxiety disorders, panic disorders, severe mood and behavior disturbances, depression, and suicide (28, 183).

Children and adolescents with indications of DTD, such as those with multiple ACEs (i.e., polyvictimization), are more likely to display suicidal behaviors and make suicide attempts than individuals without early interpersonal traumas (16). Likewise, these children may have higher rates of school absenteeism and learning disorders due to DTD (184, 185). Children with DTD are affected across cognitive (e.g., learning, attention, and information processing), physiological (e.g., fight-or-flight, susceptibility to infections including from common colds and flu), emotional (e.g., depression and suicide, anger), and behavioral domains that span the continuum of internalizing and externalizing disturbances. Behavioral displays (or traumatic reenactments) that signal fear, emotional dysregulation, and vulnerability interfere with children's academic achievement, self-efficacy, and classroom behaviors.

When individuals with DTD move into young adulthood, they may struggle to manage independently and experience difficulty adjusting to new settings. If they attend college or university, they may find it hard to self-regulate and map out the steps needed to complete assignments and tasks successfully. They may question the value of education, learning, or effort, given persistent feelings of sadness, hopelessness, and existential despair. Some may drop out of school, experience inconsistent employment, or hold employment positions that are not satisfying (167, 186), likely because their vocational identity development was interrupted by chronic depression and anxiety (16, 187, 188) resulting from reoccurring abuse and neglect. Their lack of confidence and low self-esteem, along with high rates of addiction (189–191), can also lead to further increased anxiety, anger and hostility, and job instability (192, 193). They may feel worried about re-traumatization or being stigmatized because of their challenges. As a result, some will choose not to disclose personal information to others who might help them, believing that the vulnerability could put them at a disadvantage for recruitment or advancement.

Clinicians and care supporters working with individuals who have experienced significant childhood trauma should be aware that DTD symptoms differ depending on the specific traumas experienced, the characteristics of the individuals involved, and the level of discordance between the individuals and their environments. Symptoms will also vary depending on each individual's degree of environmental support, perceived controllability of the stressful events, access to socioeconomic resources, and effective evaluations and treatments (194). Medical problems and somatic complaints may actually be the result of childhood trauma and a sign of DTD. In fact, a number of chronic, non-specific, or unexplained medical illnesses are associated with DTD (13, 56). In addition, lowered immunity, cardiac symptoms, and neurological disorders such as migraines and chronic fatigue are also signs to look for in patients who could potentially have DTD (195).

Clinicians working with early trauma survivors should also consider relational patterns corresponding to substantive behavioral changes, particularly personality disturbances. Individuals exposed to severe abuse, neglect, and violence may go on to develop long-lasting negative schemas about themselves and the world, including deep feelings of inferiority, fear, self-hatred, and distrust, all of which can give rise to maladaptive personality styles and, in some cases, clinically significant characterological disturbances (196). As adults, individuals with DTD may develop a broad range of personality and emotional disorders (60, 68, 197), which are associated with high rates of emergency room visits and inpatient hospitalizations (17, 198).

### Treatment

Empirically validated treatments for children and adolescents with DTD are limited in scope. However, there is growing evidence for integrative treatment approaches and relationally focused interventions as effective treatments for DTD [e.g., (4, 199)]. Two such approaches include the Attachment, Regulation, and Competency (ARC) model and dyadic-developmental psychotherapy, which include individual and family counseling sessions designed to enhance interpersonal communication, attachment security, and emotion regulation, and co-regulation between the parent and child; (200, 201).

Concerning adolescents and adults with trauma, findings from recent systematic investigations and meta-analyses (202–204) suggest that a combination of psychotherapy (e.g., Trauma-Focused Cognitive Behavior Therapy) and antidepressant medication (205) are beneficial. However, more studies are needed as treatment effects were heterogeneous. Likewise, more studies are required to establish the long-term efficacy and risk-to-benefit tradeoff for the use of antipsychotic agents (risperidone, quetiapine), especially for children and adolescents, as well as anticonvulsant drugs (206), which are limited in use due to their side effects.

## Conclusions and future directions

While developmental trauma is commonly linked to PTSD, the DTD diagnosis captures a more chronic and pervasive form of trauma represented by a constellation of complex and interacting cognitive, emotional, and behavioral symptoms beyond that of PTSD alone. Several studies suggest that children, adolescents, and adults with DTD display a broad range of mental health symptom profiles [e.g., (30, 31, 178, 207, 208)]. These individuals demonstrate significant problems in many areas of their lives, but without accurate assessment, advocacy, and treatment, they may live in mental anguish, vacillating between self-injury, suicidal and antisocial behaviors, and a significant inability to cope with reality and enhance their strengths and resiliencies. The DTD diagnosis offers clinicians the opportunity to more comprehensively account for the multitude of what might otherwise be viewed as divergent symptoms and discrete mental health disorders.

The DTD diagnosis offers an all-encompassing conceptualization, within a single diagnostic, developmental syndrome, of the many different symptoms that may be experienced as a result of early trauma. The diagnosis may help limit the over-pathologizing of these individuals (i.e., they would receive a single diagnosis to capture their broad array of symptoms as opposed to three or more disparate diagnoses). More importantly, utilization of the DTD framework may increase the likelihood of more accurate assessment; appropriate symptom management; well-aligned, effective treatments; and better short- and long-term outcomes. Recognition of complex trauma may provide insights that could enhance the evaluation, treatment, and prognosis for these individuals and, at the same time, build or enhance their strengths (which are likely to be undermined if a positive psychology framework does not balance a traditional, deficit-driven approach). Application of the DTD diagnosis may help decrease inappropriate and inadequate treatment approaches by using multi-system and multidisciplinary approaches when working with individuals who have suffered repeated and severe early abuse.

Consistent with Bronfenbrenner's theoretical model, an appreciation of the pervasiveness and range of opportunities to reverse the adverse effects of DTD would allow for additional, coordinated efforts among educational, legal, medical, and political leaders and stakeholders. This type of understanding will be required for substantive change to occur for individuals diagnosed with this disorder. At the organizational level, systems must promote formal policies that acknowledge, respect, and de-stigmatize DTD. According to the Substance Abuse and Mental Health Services Administration's Trauma-Informed Approach, this can be accomplished by engendering principles of neurodiversity, implementing social justice frameworks, and acknowledging the multiplicity of trauma associated with various minority statuses (209). Recommended system-wide policies and procedures include enhanced and coordinated evaluation protocols, formal response training, long-term care initiatives, and community engagement aimed at recognizing, supporting, and treating those with DTD (210–212).

In sum, research studies have established that developmental trauma confers significant risks to the health and wellbeing of those who have experienced early, repeated interpersonal traumas. Disturbances in attachment, emotion regulation, self-perception, and worldview assumptions are precipitated by trauma and cause a broad range of correlated psychological and medical disorders. Future studies are needed to reconcile several outstanding questions related to ACEs. For example, psychometric investigations of individuals who have experienced ACEs raise the possibility that additional typologies may be present [e.g., borderline personality and resilient subgroupings; (66, 213)]. Another avenue for research concerns the possibility that complex trauma is correlated with multiple sensitive periods, particularly for individuals who have been subjected to prolonged violence exposure. Lastly, although ACEs have been widely studied and consistently predict traumatic responses in adults, the construct encompasses a broad range of adversities, such as parent divorce, sexual assault, and neglect, which may confer different traumatic adaptations.

## Author contributions

DC was responsible for the conceptualization, organization, and final draft of the manuscript. All authors contributed to the literature review, writing of the manuscript, project management, and approved the final manuscript.

## Conflict of interest

The authors declare that the research was conducted in the absence of any commercial or financial relationships that could be construed as a potential conflict of interest.

## Publisher's note

All claims expressed in this article are solely those of the authors and do not necessarily represent those of their affiliated organizations, or those of the publisher, the editors and the reviewers. Any product that may be evaluated in this article, or claim that may be made by its manufacturer, is not guaranteed or endorsed by the publisher.
